# The Influence of Objectively Measured Physical Activity During Pregnancy on Maternal and Birth Outcomes in Urban Black South African Women

**DOI:** 10.1007/s10995-018-2504-3

**Published:** 2018-03-07

**Authors:** Estelle D. Watson, Søren Brage, Tom White, Kate Westgate, Shane A. Norris, Mireille N. M. Van Poppel, Lisa K. Micklesfield

**Affiliations:** 1 Centre for Exercise Science and Sports Medicine, School of Therapeutic Sciences, Faculty of Health Sciences, University of the Witwatersrand, Physical Education Building, WITS Education Campus, 27 St Andrews Rd, Parktown, Johannesburg, 2194 South Africa; 2 MRC/WITS Developmental Pathways for Health Research Unit, Department of Paediatrics, Faculty of Health Sciences, School of Clinical Medicine, University of the Witwatersrand, Private Bag X3, Wits, Johannesburg, 2050 South Africa; 3 MRC Epidemiology Unit, Institute of Metabolic Science, University of Cambridge, School of Clinical Medicine, Box 285, Cambridge Biomedical Campus, Cambridge, CB2 0QQ UK; 4 Institute of Sport Science, University of Graz, Mozartgasse 14, 8010 Graz, Austria; 5 Department of Public and Occupational Health, Amsterdam Public Health research institute, VU University Medical Center, Amsterdam, The Netherlands

**Keywords:** Physical activity, Pregnancy, Maternal outcomes, Birth outcomes, Low-to-middle income country

## Abstract

*Objectives* Research indicates the beneficial effects of physical activity during pregnancy on maternal health, although controversy still exists regarding its influence on birth outcomes. Little research has been done to objectively measure physical activity during pregnancy in black African women from low-to-middle income countries. The purpose of this study was to examine the association between physical activity and maternal and birth outcomes in this unique population. *Methods* This observational, longitudinal study assessed total physical activity using a hip-mounted triaxial accelerometer at 14–18 weeks (second trimester, n = 120) and 29–33 weeks (third trimester, n = 90) gestation. Physical activity is expressed as gravity-based acceleration units (mg). Maternal outcomes included both weight and weight gain at 29–33 weeks gestation. Birth outcomes included gestational age, birth weight, ponderal index and Apgar score, measured within 48 h of delivery. *Results* There was a significant decline in physical activity from the second to the third trimester (12.8 ± 4.1 mg vs. 9.7 ± 3.6 mg, p ≤ 0.01). Physical activity at 29–33 weeks as well as a change in PA was inversely associated with weight change at 29–33 weeks (β = − 0.24; 95% CI − 0.49; − 0.00; p = 0.05 and β = − 0.36; 95% CI − 0.62; − 0.10; p = 0.01, respectively). No significant associations were found between physical activity and birth outcomes. *Conclusions for Practice* Physical activity during pregnancy may be an effective method to control gestational weight gain, whilst presenting no adverse risk for fetal development, in women from a low-income urban setting.

## Significance

This is one of the first studies to assess patterns and effects of objectively measured physical activity on maternal and birth outcomes in Africa. In this sample of urban, black South African women we found no evidence for the effect of physical activity on birth outcomes. On the other hand, physical activity in the third trimester, and change in physical activity was inversely associated with weight change. Therefore, physical activity may be an effective method to control weight gain in this population.  

## Introduction

Current research supports the recommendation for regular physical activity (PA) during pregnancy, as it has been shown to reduce excessive gestational weight gain, and associated complications such as gestational diabetes mellitus (da Silva et al. 2016) and preeclampsia (Aune et al. 2014). Excessive gestational weight gain in particular appears to have an adverse effect on maternal health risk and cardiovascular outcomes later in life. Women with a high gestational weight gain have a greater risk of being overweight postpartum (Fraser et al. 2011), and maternal overnutrition during pregnancy may put the fetus at risk of macrosomia and later life obesity through influencing appetite and metabolism (Catalano and Ehrenberg 2006). Therefore, physical activity interventions during pregnancy may have the opportunity to break the obesity cycle in current and future generations (Catalano and Ehrenberg 2006).

Since the physiology of the woman and fetus are so closely linked, maternal physical activity may also provide health benefits for the fetus. In a systematic review, Schlüssel et al. (2008) reported that light to moderate PA during pregnancy reduces the risk of low birth weight and premature birth. However, other studies show no association between PA and birth weight (Haakstad and Bø 2011). Concerning preterm birth, it may be that the risk depends on the type of PA that is performed. In a randomised controlled trial, Barakat et al. (2008) found no association between leisure PA and gestational age, although a physically demanding job or housework may increase the risk of premature birth (Mozurkewich et al. 2000). Although women from low-to-middle income countries have a diverse way of life and PA pattern, the contribution of PA to maternal and birth outcomes has not been well explored in these populations.

Physical activity is a complex behaviour, and accurate measurement remains a challenge. Accurate PA measurement is critical to determine associations between exposure and outcomes, and it is exactly this issue that often causes such variations in findings between studies. A consistent limitation in most population studies is the use of questionnaire-based methods of measuring PA during pregnancy. These self-report measures have possibly resulted in conflicting evidence for the effect of PA on preterm birth (Schlüssel et al. 2008; Ferraro et al. 2012) and birthweight (Haakstad and Bø 2011). PA-related energy expenditure is most accurately measured through doubly-labelled water combined with indirect calorimetry of resting metabolic rate; however, these techniques are either prohibitively expensive, or are not feasible for assessing PA in a free-living environment in large scale studies (Yang and Hsu 2010). For this reason, body-worn accelerometers have become increasingly popular as a useful method for measuring PA in population studies, and in a range of settings, including during pregnancy (van Hees et al. 2011). Several studies have used accelerometers to assess levels and patterns of activity during pregnancy (Hjorth et al. 2012; Poudevigne and O’Connor 2006), and yet only a few studies have examined the association between objectively assessed PA and birth outcomes (da Silva et al. 2016; Bø et al. 2016). Many of these studies have been done in high-income countries, and there is a lack of objectively measured physical activity during pregnancy in low-to-middle income countries and even more so in Africa (Mukona et al. 2016). Therefore, the aim of the current study is to describe the level and change in objectively measured PA during pregnancy, and examine the association between PA and various maternal and birth outcomes in black South African women.

## Methods

This observational study was nested within a larger pregnancy study (N = 644) in the MRC/Wits Developmental Pathways for Health Research Unit, based at the Chris Hani Baragwanath Academic Hospital, in Soweto, South Africa. Soweto, an English abbreviation for “South Western Township”, is a large urban area of Johannesburg with the majority of inhabitants coming from low-income households. Data collection for the larger study included six time points: < 14 weeks; 14–18 weeks; 19–23 weeks; 24–28 weeks; 29–33 weeks and 34–38 weeks gestation. For this study, data from baseline (< 14 weeks gestation) was used, and the sub-study was defined as all participants with valid PA measurements at 14–18 weeks and 29–33 weeks gestation (to allow for the measurement of PA in both the second and the third trimester, as well as change in PA during the course of pregnancy). Women with a singleton pregnancy, attending the hospital for antenatal care, were recruited into the sub-study between May 2014 and August 2015, and the participant number was based on the amount of accelerometers available at the time of the visit. Participants were provided with an information sheet which described the study, including the risks and benefits of taking part, and all participants voluntarily signed the consent form prior to participation. The sub-study was approved by the Human Research Ethics Committee of the University of the Witwatersrand (Clearance number M130351).

All demographic and anthropometric data were collected by experienced and trained research assistants/nurses. Demographic and socioeconomic information was gathered using an interviewer-led questionnaire. Socioeconomic status was assessed using household inventory questions assessing the ownership of nine household commodities (electricity, radio, television, refrigerator, cell phone, personal computer, bicycle, motorcycle/scooter, car). In addition, household density was calculated as the number of people living in the household divided by the number of rooms used for sleeping. All demographic, socioeconomic and behavioural data were collected at the baseline visit (< 14 weeks gestation).

Physical activity was measured using a hip-worn triaxial accelerometer (ActiGraph GT3X+, ActiGraph, Pensacola, FL), at two time points during pregnancy (14–18 weeks and 29–33 weeks gestation). The device was initialized to record at a sample rate of 30 Hz, for seven consecutive days. Participants were instructed to remove the device when washing or bathing, and during sleep. Non-wear time was defined as periods lasting 3 h or longer where the standard deviation of acceleration in each axis remained below 5 mg (van Hees et al. 2014). All data from midnight to 6am were excluded, regardless of being classified as the device being worn. A day was considered valid if it contained at least 7 h of wear time, and a minimum of 3 valid days of wear time was required for a record to be included in this analysis. Acceleration was calibrated to local gravity (van Hees et al. 2014) and expressed in gravity-based acceleration units (mg), following which a measure of overall PA volume was derived from the acceleration data using the metric Euclidean Norm Minus One (ENMO) statistic (van Hees et al. 2014). This is the vector magnitude of acceleration in all three axes, and subtracting the value of gravity (x^2^ + y^2^ + z^2^)^½^ − 1, with any negative values rounded up to zero. The use of ENMO for interpreting and estimating PA is well supported and has been used in other studies (White et al. 2016; van Hees et al. 2014; da Silva et al. 2014).

Anthropometric measurements: height (cm) was measured using a stadiometer (SECA, Hamburg, Germany), and body weight (kg) was measured to the nearest 0.1 kg using a digital weighing scale (SECA, Hamburg, Germany). Body mass index was calculated at baseline (< 14 weeks) as weight (kg)/height (m)^2^, and classified according to the World Health Organization’s categories for underweight (< 18.5 kg/m^2^), normal weight (≥ 18.5–24.9 kg/m^2^), overweight (≥ 25–29.9 kg/m^2^) and obese (≥ 30 kg/m^2^). Absolute weight (kg) at 29–33 weeks was used as an outcome, and gestational weight change was calculated as the difference between weight at baseline (< 14 weeks) and weight at the 29–33 weeks visit.

Gestational age (in weeks) was accurately determined by using ultrasound measurements taken by research sonographers at the baseline visit. Trained research nurses collected birth outcomes and neonatal anthropometric data (birth weight, length, and head circumference) within 48 h of delivery. Initially missing data was back-filled with information from hospital delivery records. Ponderal index was calculated as birth weight (g)/height (cm)^3^. Apgar score at the time of birth was collected from hospital delivery records.

Descriptive data is presented as mean ± standard deviation (SD) for parametric data and n(%) for non-parametric data. Imputation was used for missing PA values in the third trimester (29–33 weeks), using linear regression of PA in the 3rd trimester on PA in the 2nd trimester. The PA 3rd trimester variable used in the models is a consolidated variable containing both measured and imputed data, and a separate variable indicating measurement status (measured/imputed) was added as a co-variate in the multiple linear regression models. Non-normally distributed data (PA at 14–18 weeks, PA at 29–33 weeks, BMI, Ponderal Index) was log-transformed prior to analysis. Since PA data remained non-normally distributed after log transformation, a Wilcoxon Signed Rank test was performed to compare PA measures between the two time points. Multiple linear regression analyses were performed to determine the exposure variables associated with maternal outcomes, namely absolute weight (at 29–33 weeks) and weight change (from < 14 weeks to the 29–33 weeks’ time point), in addition to neonatal outcomes (gestational age, birth weight, ponderal index, Apgar score). The models were adjusted for a combination of baseline BMI, age, imputed data, days from baseline to 29–34 weeks (continuous data) and parity (having had a previous child), education (having secondary education or less), smoking (currently smoking during this pregnancy), and/or HIV status (being HIV positive). The models were not adjusted for those women with Gestational Diabetes Mellitus (n = 4) due to the small sample size. Analyses were done using Stata (Version 11; StataCorp LP), and statistical significance was set at p ≤ 0.05.

## Results

**Fig. 1:**
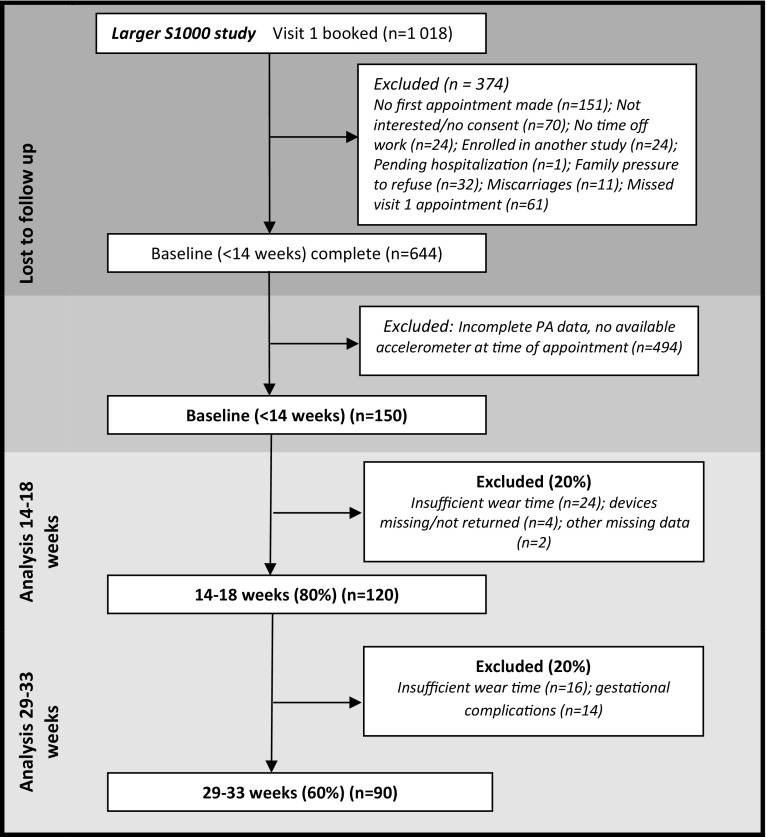
Participant flow through the study

A total of 150 women were initially recruited into the sub-study, and 120 (80%) had valid activity data at 14–18 weeks gestation, and 90 (60%) had valid activity data at 29–33 weeks gestation. Ten women had valid PA data at 29–33 weeks, but not at 14–18 weeks, making the total sample n = 130. At 14–18 weeks gestation, reasons for exclusion included insufficient accelerometer wear time (n = 24), devices missing/not returned (n = 4) and other missing data (n = 2). At 29–33 weeks gestation, 16 women had delivered prior to their 29–33 week visit, 4 women had miscarriages, 2 women were admitted to hospital for gestational complications, 22 women were lost to follow up. An additional 16 women were excluded due to insufficient wear time (Fig. 1). No differences in age (p = 0.97) or second trimester PA (p = 0.67) were found between those who were lost to follow up and those who remained in the study; however the group lost to follow up had higher baseline BMI (p = 0.02).

Maternal demographic, anthropometric and behavioural characteristics, in addition to maternal and birth outcomes, of the 130 women who participated in the study, are presented in Table 1. A high prevalence of overweight (40.8%) and obesity (28.5%) was found at baseline (< 14 weeks gestation), and 28.4% of the women were HIV positive by self-report. The majority of women had completed secondary schooling (70.5%) and owned 5–7 items on the household inventory (77.7%).

**Table 1:** Description of sociodemographics, and behavioural characteristics and outcomes of the participants

	n	Mean ± SD or n (%)
Anthropometrics < 14 weeks		
Age (years)	130	30.4 ± 5.8
Height (cm)	130	158.8 ± 5.9
Baseline weight (kg) < 14 weeks	130	69.9 ± 14.2
BMI (kg/m^2^) < 14 weeks	130	27.7 ± 5.2
BMI classification	130	
Underweight		2 (1.5%)
Normal weight		38 (29.2%)
Overweight		53 (40.8%)
Obese class I		37 (28.5%)
Sociodemographic (< 14 weeks)		
Marital status	127	
Single		73 (57.5%)
Married/cohabiting		54 (42.5%)
Level of education	129	
Completed primary school		5 (3.9%)
Completed secondary school		91 (70.5%)
Completed tertiary education		33 (25.6%)
Employment status	130	
Manual work		32 (24.8%)
Non-manual work		29 (22.5%)
Unemployed		33 (25.6%)
Other		34 (26.4%)
Household inventory	130	
Low (< 5)		7 (5.4%)
Medium (5–7)		101 (77.7%)
High (> 7)		22 (16.9%)
Behavioural (< 14 weeks)		
Smoking or chewing tobacco/betelnut?	128	
Yes		12 (9.4%)
No		116 (90.6%)
Physical activity levels		
Physical activity (mg) 14–18 weeks gestation	120	12.8 ± 4.1
Physical activity (mg) 29–33 weeks gestation	90	9.7 ± 3.6^a^
Change in physical activity	81	-3.1 ± 4.5
Maternal outcomes		
Weight (kg) 29–33 weeks gestation	108	76.4 ± 13.8
Weight gain (kg) 29–33 weeks gestation	108	7.5 ± 4.5
Birth outcomes		
Gestational age (weeks)	126	36.2 ± 4.6
Gestational age classification	121	
Premature (< 37 weeks)		50 (41.3%)
Term (≥ 37 weeks)		71 (58.7%)
Newborn weight (g)	120	2876.1 ± 647.0
Newborn weight classification	120	
Low birth weight (< 2500 g)		30 (25.0%)
Normal (> 2500 g)		88 (73.3%)
Macrosomia (> 4500 g)		2 (1.7%)
Newborn weight classification	118	
Small for gestational age (< 10th percentile)		16 (13.6%)
Appropriate for gestational age (10th–90th percentile)		96 (81.4%)
Large for gestational age (> 90th percentile)		6 (5.1%)
Newborn length (cm)	119	47.2 ± 5.1
Newborn head circumference (cm)	119	34.3 ± 2.8
Ponderal index (g/cm^3^)	119	2.8 ± 0.8
Apgar score (5 min)	118	9.7 ± 0.7
Pregnancy outcome	129	
Live born		100 (77.5%)
Live born, with complications/diagnosed condition		17 (13.2%)
Antepartum death		6 (4.7%)
Neonatal death		6 (4.7%)

^a^p<0.01 for PA 14–18 weeks versus 29–33 weeks (mg, gravity-based acceleration)

The accelerometer was worn for a mean of 6.4 ± 0.9 days at 14–18 weeks and 6.0 ± 1.2 days at 29–33 weeks for those included in the final analysis. No significant difference was found in wear time between the two trimesters (p = 0.99). A significant decline (24%) in overall volume of PA was found from the second to the third trimester (12.8 ± 4.1 vs. 9.7 ± 3.6 mg, p < 0.001) (Table 1).

No association was found between PA at either time point and absolute weight at 29–33 weeks, after adjusting for early pregnancy BMI, age, parity, education, smoking and follow-up duration (from baseline to 29–33 weeks) in a multiple regression analysis (Table 2). Similarly, PA at 14–18 weeks gestation was not associated with weight change at 29–33 weeks gestation, however, PA at 29–33 weeks gestation was inversely associated with weight change at 29–33 weeks gestation (β = − 0.24; 95% CI − 0.49; − 0.00; p = 0.05). Change in PA was also inversely associated with weight change at 29–33 weeks (β = − 0.36; 95% CI − 0.62; − 0.10; p = 0.01).

**Table 2:** Multiple linear regression analysis for the association between maternal physical activity and maternal outcomes

Exposure variable	Outcome variable							
	Unadjusted model				Adjusted model			
	β (95% CI)	SE	p-Value	Adj R^2^	β (95% CI)	SE	p-Value	Adj R^2^
Absolute weight (29–33 weeks)								
PA 2nd trimester	− 12.77 (− 21.0; − 4.53)	4.14	0.01*	0.07	− 2.29 (− 6.94; 2.34)^a^	2.33	0.32	0.75
PA 3rd trimester	− 0.45 (− 1.28; 0.36)	0.41	0.27	0.01	− 0.28 (− 0.72; 0.16)^b^	0.22	0.20	0.76
Change in PA	− 0.19 (− 0.40; 0.02)	0.10	0.08	0.01	− 0.33 (− 0.80; 0.13)^c^	0.23	0.15	0.78
Weight change (< 14 weeks to 29–33 weeks)								
PA 2nd trimester	1.03 (− 1.69; 3.77)	1.37	0.45	− 0.01	1.26 (− 1.27; 3.81)^a^	1.28	0.32	0.17
PA 3rd trimester	− 0.14 (− 0.41; 0.12)	0.13	0.29	0.01	− 0.24 (− 0.49; − 0.00)^b^	0.12	0.05*	0.21
Change in PA	− 0.19 (− 0.40; 0.2)	0.10	0.08	0.02	− 0.36 (− 0.62; − 0.10)^c^	0.12	0.01*	0.19

*Indicates statistical significance (p ≤ 0.05)

^a^Adjusted for BMI (< 14 weeks), age, parity, education, smoking, HIV status, days from baseline to 29–34 weeks

^b^Adjusted for BMI (< 14 weeks), age, parity, education, smoking, HIV status, days from baseline to 29–33 weeks, imputed PA data

^c^Adjusted for BMI (< 14 weeks), age, parity, education, smoking, PA (19–23 weeks), HIV status, days from baseline to 29–33 weeks

None of the PA variables contributed significantly to any of the birth outcomes including gestational age, birth weight, Ponderal index and Apgar score (Table 3).

**Table 3:** Multiple linear regression analysis for the association between maternal physical activity and birth outcomes

Exposure variable	Outcome variable							
	Unadjusted model				Adjusted model			
	β (95% CI)	SE	p-Value	Adj R^2^	β (95% CI)	SE	p-Value	Adj R^2^
Gestational age (weeks)								
PA 2nd trimester	1.36 (− 1.31; 4.05)	1.35	0.31	0.01	0.87 (− 1.61; 4.12)^a^	1.44	0.38	− 0.01
PA 3rd trimester	0.07 (− 0.19; 0.35)	0.13	0.57	− 0.01	0.09 (− 0.14; 0.15)^b^	0.13	0.57	0.08
Change in PA	− 0.10 (− 0.26; 0.05)	0.08	0.20	0.01	0.08 (− 0.12; 0.30)^c^	0.10	0.40	0.02
Birth weight (g)								
PA 2nd trimester	37.96 (− 362.81; 438.73)	202.18	0.85	− 0.01	99.57 (− 193.99; 393.13)^d^	147.91	0.50	0.54
PA 3rd trimester	− 2.48 (− 41.53; 36.56)	19.71	0.90	− 0.01	− 7.87 (− 35.84; 20.10)^e^	14.10	0.57	0.55
Change in PA	− 19.82 (− 47.48; 7.83)	13.88	0.16	0.01	− 16.62 (− 47.97; 14.75)^f^	15.71	0.29	0.33
Ponderal index (g/cm^3^)								
PA 2nd trimester	− 0.07 (− 0.20; 0.05)	0.06	0.25	0.01	− 0.04 (− 0.17; 0.08)^d^	0.06	0.51	0.16
PA 3rd trimester	− 0.00 (− 0.02; 0.00)	0.00	0.12	0.01	− 0.00 (− 0.02; 0.00)^e^	0.00	0.22	0.14
Change in PA	− 0.00 (− 0.01; 0.00)	0.00	0.63	− 0.01	− 0.00 (− 0.02; 0.00)^f^	0.00	0.32	0.20
Apgar score								
PA 2nd trimester	0.26 (− 0.16; 0.70)	0.22	0.22	0.01	0.00 (− 0.02; 0.02)^d^	0.01	0.97	0.17
PA 3rd trimester	0.00 (− 0.03; 0.04)	0.02	0.80	− 0.01	− 0.00 (− 0.02; 0.03)^e^	0.01	0.82	0.17
Change in PA	− 0.01 (− 0.03; 0.00)	0.01	0.19	0.01	− 0.00 (− 0.03; 0.02)^f^	0.01	0.90	− 0.03

^a^Adjusted for maternal age, BMI (< 14 weeks), smoking, education and HIV status

^b^Adjusted for maternal age, BMI (< 14 weeks), smoking, education, HIV status and imputed data

^c^Adjusted for maternal age, BMI (< 14 weeks), smoking, education, HIV status and PA (14–18 weeks)

^d^Adjusted for maternal age, BMI (< 14 weeks), education, HIV status, smoking and gestational age

^e^Adjusted for maternal age, BMI (< 14 weeks), education, HIV status, smoking, gestational age and imputed data

^f^Adjusted for maternal age, BMI (< 14 weeks), education, HIV status, smoking, gestational age and PA (14–18 weeks)

## Discussion

The main findings of this longitudinal study indicate that there is a significant reduction in PA during the gestational period in a sample of black women from Soweto, South Africa with a high prevalence of overweight/obesity and HIV. Furthermore, this change in PA may play a role in weight management of pregnant woman but we found no evidence to suggest an effect of PA on birth outcomes including duration of gestation and size of the newborn baby. To our knowledge, it is the first longitudinal study to objectively measure free-living PA in black South African women during pregnancy. Incorporation of the objective measurement of biomechanical signals (raw accelerometry) has several advantages over the use of questionnaires and self-reported PA previously used (Hjorth et al. 2012), such as the avoidance of recall bias. Furthermore, few studies have assessed the effects of total PA on fetal growth and development (Schlüssel et al. 2008), and this study collected PA data at two time points during pregnancy, allowing for a longitudinal analysis of PA behaviour change and its effects on maternal and birth outcomes.

Previous studies examining the change in PA during pregnancy have reported conflicting results and methodological variations, with the use of both self-report and accelerometry to measure PA. Although some studies using self-report (Liu et al. 2011) and accelerometry (Stein et al. 2003) have reported no change in PA during pregnancy, the evidence is accumulating for declining levels of PA during pregnancy. Evenson and Wen (2011) reported significantly lower levels of moderate PA in the third trimester when compared to the first two trimesters. Similarly, Rousham et al. (2006) found a significant decline in total PA measured by accelerometry at 38 weeks when compared to 16 and 25 weeks gestation. The current study found a significant reduction in total PA from the second to the third trimester, and provides supportive evidence for the decline in PA levels during pregnancy that has been found in other studies (Hjorth et al. 2012; Evenson and Wen 2011; Rousham et al. 2006; Hayes et al. 2015). However, many of these studies assessed changes specifically in moderate-vigorous PA during pregnancy (Evenson and Wen 2011). In a study of 140 obese pregnant women, Hayes et al. (2015) found a reduction in moderate-vigorous PA from 16–18 weeks to 35–36 weeks gestation, but no change in total PA. In contrast, our study found a reduction in the total volume of PA, which is supported by the findings of Di Fabio et al. (2015) who attributed this decline in PA to shifts from moderate-to-vigorous PA to light intensity PA combined with an increase in sedentary behaviours during pregnancy. Encouragingly, there is evidence for the benefits of total PA, not exclusively moderate-to-vigorous PA, during pregnancy to reduce gestational weight gain (Cohen and Koski 2013) and increase insulin sensitivity (Hayes et al. 2015).

Societies undergoing the nutritional transition are demonstrating significant shifts in diet and PA patterns, and measures of socioeconomic status such as income and higher education, have been associated with adopting these new patterns (Popkin 1994). Although all the women in the current study were recruited from a public hospital (a proxy for low to middle socioeconomic status in South Africa), 25.6% had tertiary education and 16.9% had more than 7 household commodities, indicating that this particular sub-sample may be on the higher end of Soweto’s socioeconomic scale. In South Africa, urbanized individuals with higher educational levels have been found to consume energy-dense diets that are high in fat (Steyn and Mchiza 2014), and this may be supported by our study’s overweight and obesity prevalence (69%) during early pregnancy, that was 25% higher than other reports in black South African pregnant women (Basu et al. 2010). Of concern is that obese pregnant women face twice the risk of birth complications and gestational diabetes (Basu et al. 2010; Siega-Riz et al. 2009), in addition to postpartum maternal weight retention (Fraser et al. 2011). Furthermore, an obese pregnant population has consequences for future generations, as an increased maternal body weight has been associated with increased fetal growth and large-for-gestational-age infants (Catalano and Ehrenberg 2006).

Maternal overnutrition, and excessive gestational weight gain, have both been associated with adverse pregnancy outcomes, such as impaired glucose tolerance, gestational hypertension and gestational diabetes mellitus (Siega-Riz et al. 2009). Guidelines for the recommended ranges of gestational weight gain is provided by the Institute of Medicine (IOM), however, a study by Chasan-Taber et al. (2008) found that overweight women are more likely to gain weight above these recommendations. Furthermore, the applicability of the guidelines to obese women remains controversial, with favourable outcomes having been found in obese women that gain no weight, or below the recommendations (Kiel et al. 2007). Our study showed an inverse relationship between PA at 29–33 weeks and gestational weight change, providing evidence for the effectiveness of total PA during pregnancy in controlling gestational weight gain. This finding is supported by a meta-analysis of 12 randomised controlled trials which showed that exercise was associated with 0.61 kg (95% CI − 1.17, − 0.06) less gestational weight gain in the intervention versus the control groups (Streuling et al. 2011). Additionally, a change in PA during pregnancy was also inversely associated with weight change, implying that reducing PA during pregnancy may result in greater weight gain, whilst increasing PA levels may help to manage this increase in weight.

Despite 30 years of research on this topic, the influence of PA on fetal growth and development is inconclusive. Some research has shown no effect of PA on gestational age, whilst others have found an inverse association (Schlüssel et al. 2008; Barakat et al. 2008; Ferraro et al. 2012). A recent study assessing the effects of long term PA found that active women have lower rates of preterm birth (Vamos et al. 2015). The association between PA and birth weight follows a similar, conflicting pattern (Schlüssel et al. 2008; Ferraro et al. 2012). Vamos et al. (2015) found that the association between PA and birth weight is diminished when adjusting for sociodemographic and other confounders. The population in this study had unique co-founding factors such as high BMI levels at baseline and high HIV rates, which may dominate PA in their influence on birth outcomes, explaining the lack of evidence for an association between total PA and birth outcomes in this population.

The data in this study should be considered in light of some limitations. Many previous studies have looked exclusively at moderate-vigorous PA and not total volume of PA, making comparisons between our population and other studies difficult. Our study did not analyse intensity and type of PA, and these may have some influence on maternal and birth outcomes (Schlüssel et al. 2008). However, total volume of PA is emerging as an independent and influential factor in pregnancy outcomes (Gradmark et al. 2011), and using objective measures for assessing this is valuable when examining a dose–response relationship. Furthermore, the observational nature of the study, as well as the lack of continuous observation, makes it difficult to determine a causal relationship between the variables, and further research should include well-designed randomised controlled studies to determine the effect of PA on maternal and birth outcomes in this population. Observational accelerometer studies also have the potential to cause a hawthorne effect, whereby participants change their behaviours due to being monitored by an accelerometer. In addition, the presence of co-morbidities (such as gestational diabetes mellitus, hypertension and gestational complications) may affect both maternal and birth outcomes, however prevalence of most of these was too low and unlikely to influence the study results. Limited resources in our study setting did not allow for objective PA monitoring on a large scale, as has been done in other countries (Evenson and Wen 2011), therefore may limit the generalisability of the results to the larger population. Despite these limitations, this study is the first of its kind to objectively assess physical activity during pregnancy, and its effect on maternal and birth outcomes in a unique South African population.

## Conclusion

In summary, total volume of PA significantly decreased from the second to the third trimester and this study highlights the need for future efforts to be directed at increasing, or maintaining, PA during pregnancy. In addition, total PA in the third trimester, and increasing PA levels, was associated with a reduction in gestational weight gain. We therefore support the evidence that “every little movement counts” (Ferraro et al. 2012) and future recommendations should encourage any physical activity in this population. Lastly, our study did not provide sufficient evidence for an association between PA and birth outcomes. Thus, this study supports the conclusion that PA is beneficial for maternal outcomes, whilst posing no adverse risk to the growing fetus. In a population where the majority of pregnant women are overweight and obese, PA interventions are needed to control gestational weight gain and minimise the potential effects of obesity on future generations.
